# Data on the mechanical properties of recycled wind turbine blade composites

**DOI:** 10.1016/j.dib.2018.05.008

**Published:** 2018-05-09

**Authors:** Seyed Hossein Mamanpush, Azadeh Tavousi Tabatabaei, Hui Li, Karl Englund

**Affiliations:** Composite Materials and Engineering Center, Washington State University, Pullman, WA, 99163, USA

## Abstract

Wind turbine blades that face end-of-life recycled mechanically. The recycled material was first comminuted via a hammer-mill through a range of varying screen sizes, resonated (polymeric Methylene diphenyl isocyanate (pMDI)) and then hand-formed and hot pressed. The hot press temperature and time were set as 138 °C and 5 min accordingly, typical for pMDI composite processing. Mechanical properties (Modulus of rupture (MOR), Module of elasticity (MOE) and Internal bond(IB)) dataset of composites made from recycled wind turbine blades(rWTBs) presented. Dataset also presented the influence of resin level, moisture content, mill screen size and density on the mechanical properties of composites [1], [2].

**Specifications Table**

**Table:** 

Subject area	Composite material
More specific subject area	Recycling thermoset composite material
Type of data	Table
How data was acquired	Mechanical properties are obtained by experimental method using ASTM 1037-12
Data format	Raw, Analyzed
Experimental factors	
Experimental features	
Data source location	Composites Materials and Engineering Center at Washington State University
Data accessibility	Data are accessible in this article.
Related research article	Seyed Hossein Mamanpush, et.al., Recycled wind turbine blades as a feedstock for second generation composites [1]

**Value of the data**

• Presented dataset shows consistency among the samples and helps researcher to see the actual trend among these second generation composites with different formulation.
• Raw dataset presented on mechanical properties of rWTB composites helps other researcher in this field to understand the original condition of these second generation composites.
• Data on Mechanical properties of second generation composites manufactured from rWTB materials gives the researchers clear vision about the potential utilization of these second generation composites.
• Based on presented data, researchers could be referred to this dataset to design and analyze different experiments on rWTBs.

## 1 Data

For obtaining mechanical properties of composites fabricated using recycled wind turbine blade materials, flexural and Internal bond tests were performed based on ASTM D1037-12. Presented dataset include influence of resin level (MDI (%)), moisture content (MC(%)), mill screen size (MSS(mm)) and density on the mechanical properties of second generation composites.

## 2 Experimental design, materials, and methods

### 2.1 Materials

Recycled wind turbine blade (rWTB) material supplied by Global Fiberglass Solutions at an incoming MC of 1.25%. A polymeric methyl-diisocyanate (Rubinate 1840) (pMDI) resin was kindly supplied by Huntsman and was used as the binder for the second generation panel product. The rWTB material was then hammer-milled through 12.7, 6.35, 3.18, and 1.59 mm screen size, respectively.

### 2.2 Manufacturing of rWTB composites

**Fig. 1:**
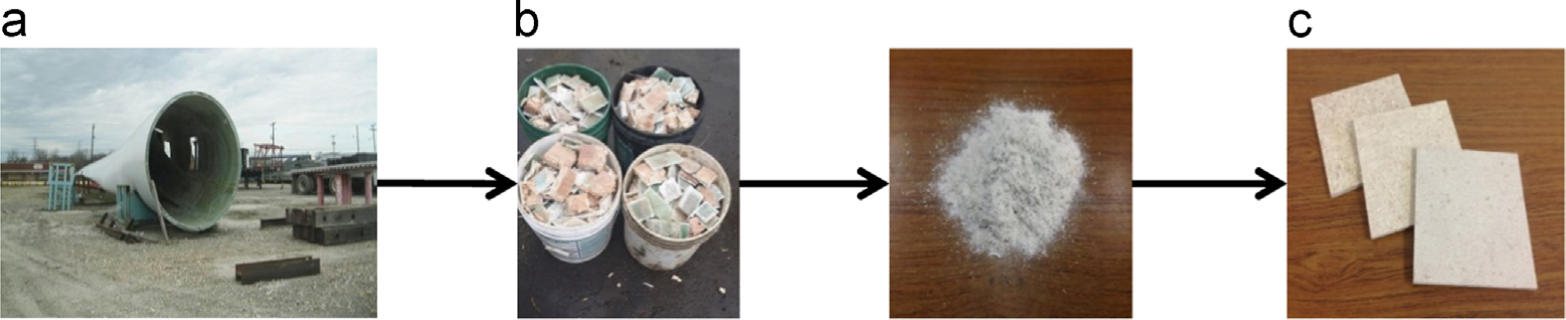
(a) End-of-life wind turbine blade, (b) mechanical comminution process, (c) hot pressed rWTB composites [1].

The various size fractions of the rWTB materials were sprayed with resin (3,6 and 10%) and water (to obtain the targeted MC (3, 5 and 8%)) within a drum blender. The blended rWTB was then hand-formed and hot pressed to a size of 355.6×355.6mm *composites panels (duplicate) with a thickness of 7.62 mm (*Fig. 1*).*

### 2.3 Mechanical properties

Datasets demonstrating physical condition of each specimen prepared for Flexural and IB test are as follow:

#### 2.3.1 Flexural and IB test

The rWTB composites with 10MDI, 5MC and 12.7 mm MSS has the maximum amount of MOE and is equal to 5254 MPa and the rWTB composites with 1.59 mm MSS, 6MDI and 5 MC has the minimum amount of MOE equal to 2910 MPa.

Similar to MOE, rWTB composites with 10MDI and 5MC has the maximum amount for both of the MOR and IB that are 41.6 MPa and 2.35 MPa respectively. The rWTB composites with 1.59 mm MSS has the minimum amount of MOR equal to 23.8 MPa and composite with 3MDI, 5MC and 12.7 mm MSS has the minimum amount of IB equal to 0.82 MPa. The best results for IB is for 1.59 mm MSS (Table 1, Table 2, Table 3, Table 4).

**Table 1:** physical properties of each specimen prepared for Flexural test.

Sample formulation														Average	
MDI (%)	MC (%)		MSS (mm)	Width(mm)					Depth (mm)					Width (mm)	Depth (mm)
10	5		12.7	75.95	76.08	76.046	7.378	7.378	7.378	7.378	7.378	7.378	7.378	7.378	7.378
10	5		12.7	76.38	76.29	76.214	7.312	7.312	7.312	7.312	7.312	7.312	7.312	7.312	7.312
10	5		12.7	76.35	76.37	76.34	7.516	7.516	7.516	7.516	7.516	7.516	7.516	7.516	7.516
10	5		12.7	76.36	76.09	76.078	7.186	7.186	7.186	7.186	7.186	7.186	7.186	7.186	7.186
10	5		12.7	76.26	76.28	76.342	7.224	7.224	7.224	7.224	7.224	7.224	7.224	7.224	7.224
10	5		12.7	76.39	76.29	76.294	7.466	7.466	7.466	7.466	7.466	7.466	7.466	7.466	7.466
3	5		12.7	76.04	76.26	76.152	7.04	7.04	7.04	7.04	7.04	7.04	7.04	7.04	7.04
3	5		12.7	76.24	76.2	76.25	7.518	7.518	7.518	7.518	7.518	7.518	7.518	7.518	7.518
3	5		12.7	76.35	76.11	76.224	7.508	7.508	7.508	7.508	7.508	7.508	7.508	7.508	7.508
3	5		12.7	76.23	76.15	76.204	7.656	7.656	7.656	7.656	7.656	7.656	7.656	7.656	7.656
3	5		12.7	76.1	76.11	76.114	7.872	7.872	7.872	7.872	7.872	7.872	7.872	7.872	7.872
3	5		12.7	76.36	76.2	76.23	7.77	7.77	7.77	7.77	7.77	7.77	7.77	7.77	7.77
6		3	12.7	76.11	76.18	76.224	7.264	7.264	7.264	7.264	7.264	7.264	7.264	7.264	7.264
6		3	12.7	76.2	76.3	76.288	7.314	7.314	7.314	7.314	7.314	7.314	7.314	7.314	7.314
6		3	12.7	75.98	75.97	75.966	7.63	7.63	7.63	7.63	7.63	7.63	7.63	7.63	7.63
6		3	12.7	76.02	76.11	76.092	7.688	7.688	7.688	7.688	7.688	7.688	7.688	7.688	7.688
6		3	12.7	75.96	75.95	75.958	7.72	7.72	7.72	7.72	7.72	7.72	7.72	7.72	7.72
6		3	12.7	75.85	75.96	76.004	7.656	7.656	7.656	7.656	7.656	7.656	7.656	7.656	7.656
6		8	12.7	76.12	75.95	75.96	7.442	7.442	7.442	7.442	7.442	7.442	7.442	7.442	7.442
6		8	12.7	75.87	75.92	75.968	7.42	7.42	7.42	7.42	7.42	7.42	7.42	7.42	7.42
6		8	12.7	75.75	75.77	75.748	7.262	7.262	7.262	7.262	7.262	7.262	7.262	7.262	7.262
6		8	12.7	75.76	75.78	75.862	7.04	7.04	7.04	7.04	7.04	7.04	7.04	7.04	7.04
6		8	12.7	76.01	75.97	75.99	6.926	6.926	6.926	6.926	6.926	6.926	6.926	6.926	6.926
6		5	3.18	76.16	76.16	76.094	7.59	7.59	7.59	7.59	7.59	7.59	7.59	7.59	7.59
6		5	3.18	76.04	76.05	76.084	7.384	7.384	7.384	7.384	7.384	7.384	7.384	7.384	7.384
6		5	3.18	75.76	75.71	75.726	7.438	7.438	7.438	7.438	7.438	7.438	7.438	7.438	7.438
6		5	3.18	75.75	75.61	75.67	7.464	7.464	7.464	7.464	7.464	7.464	7.464	7.464	7.464
6		5	3.18	75.51	75.59	75.588	7.472	7.472	7.472	7.472	7.472	7.472	7.472	7.472	7.472
6		5	3.18	75.68	75.62	75.604	7.436	7.436	7.436	7.436	7.436	7.436	7.436	7.436	7.436
6		5	0.25	76.32	76.3	76.22	7.032	7.032	7.032	7.032	7.032	7.032	7.032	7.032	7.032
6		5	0.25	76.31	76.32	76.3	7.246	7.246	7.246	7.246	7.246	7.246	7.246	7.246	7.246
6		5	0.25	76.04	75.95	75.982	7.59	7.59	7.59	7.59	7.59	7.59	7.59	7.59	7.59
6		5	0.25	76.1	76.11	76.17	7.588	7.588	7.588	7.588	7.588	7.588	7.588	7.588	7.588
6		5	0.25	76.25	76.17	76.124	7.692	7.692	7.692	7.692	7.692	7.692	7.692	7.692	7.692
6		5	0.25	76.3	76.26	76.224	7.612	7.612	7.612	7.612	7.612	7.612	7.612	7.612	7.612
6		5	0.125	76.14	76.15	76.184	7.662	7.662	7.662	7.662	7.662	7.662	7.662	7.662	7.662
6		5	0.125	76.28	76.19	76.186	7.732	7.732	7.732	7.732	7.732	7.732	7.732	7.732	7.732
6		5	0.125	75.52	75.51	75.53	7.314	7.314	7.314	7.314	7.314	7.314	7.314	7.314	7.314
6		5	0.125	75.74	75.87	75.926	7.24	7.24	7.24	7.24	7.24	7.24	7.24	7.24	7.24
6		5	0.125	75.61	75.78	75.884	7.138	7.138	7.138	7.138	7.138	7.138	7.138	7.138	7.138
6		5	0.125	75.78	75.83	75.926	7.068	7.068	7.068	7.068	7.068	7.068	7.068	7.068	7.068

**Table 2:** physical properties of each specimen prepared for IB test.

Sample formulation			Weight (g)	Height (mm)	Width (mm)	Thickness (mm)	Volume (mm^3)	Density (g/cm^3)
MDI (%)	MC (%)	MSS (mm)						
10	5	12.7	18.82	50.82	50.81	7.08	18281.72254	1.029443476
10	5	12.7	19.9	50.98	50.67	7.09	18314.58029	1.086565986
10	5	12.7	19.91	50.55	50.83	7.09	18217.44659	1.092908378
10	5	12.7	17.56	50.22	50.86	7.16	18287.99467	0.960192756
10	5	12.7	18	50.46	50.91	7.19	18470.52473	0.974525643
10	5	12.7	20.5	50.4	50.94	7.29	18716.17104	1.095309503
3	5	12.7	18.94	50.93	51.1	7.06	18373.81238	1.030814923
3	5	12.7	18.1	50.76	50.83	7.2	18576.94176	0.974326142
3	5	12.7	19.34	50.87	50.81	7.39	19100.96773	1.012514144
3	5	12.7	19.39	50.7	50.85	7.2	18562.284	1.04459128
3	5	12.7	19.2	50.66	50.95	7.35	18971.28345	1.012055934
3	5	12.7	19.39	50.52	51.05	7.33	18904.40718	1.025686752
6	3	12.7	19.23	50.93	50.93	7.53	19531.8027	0.984548139
6	3	12.7	18.19	50.76	50.85	7.44	19203.72624	0.947212003
6	3	12.7	19.44	50.83	50.66	7.65	19699.11567	0.98684633
6	3	12.7	19.61	50.75	50.84	7.7	19867.001	0.987063926
6	3	12.7	20.03	50.81	50.78	7.54	19454.19377	1.029598051
6	3	12.7	19.02	50.45	50.9	7.56	19413.3618	0.979737574
6	8	12.7	21.24	50.49	51.03	7.36	18963.07459	1.120071531
6	8	12.7	19.71	50.3	50.95	7.26	18605.8191	1.059345998
6	8	12.7	20.06	50.18	51	7.29	18656.4222	1.075232957
6	8	12.7	19.38	50.1	51	7.29	18626.679	1.040443119
6	8	12.7	19.93	50.11	50.98	7.17	18316.53793	1.088087721
6	5	3.18	19.42	50.96	50.95	7.59	19706.76708	0.985448294
6	5	3.18	20.07	51.02	50.86	7.62	19772.96426	1.015022317
6	5	3.18	19.86	50.99	50.82	7.69	19927.18774	0.996628338
6	5	3.18	21.08	51.04	50.81	7.61	19735.33566	1.06813486
6	5	3.18	21.38	50.8	50.99	7.66	19841.63672	1.077532076
6	5	3.18	19.97	50.89	50.84	7.54	19507.8469	1.023690626
6	5	0.25	18.98	51.04	50.98	7.52	19567.18438	0.969991371
6	5	0.25	19.37	50.88	50.85	7.59	19637.21232	0.986392553
6	5	0.25	20.42	50.84	50.84	7.64	19747.15078	1.03407323
6	5	0.25	21.89	50.84	50.92	7.61	19700.56101	1.11113587
6	5	0.25	22.01	50.87	50.91	7.48	19371.64192	1.136196926
6	5	0.25	20.39	50.96	50.94	7.34	19053.92362	1.070120801
6	5	0.125	18.68	50.74	50.94	7.19	18583.96136	1.005167824
6	5	0.125	18.42	50.79	50.73	7.17	18474.05494	0.997074008
6	5	0.125	17.69	50.66	50.8	7.18	18477.93104	0.957358265
6	5	0.125	18.09	50.73	50.77	7.24	18647.0696	0.970125622
6	5	0.125	17.93	50.78	50.75	7.26	18709.6371	0.958329651
6	5	0.125	18.55	50.71	50.81	7.32	18860.52973	0.983535471

**Table 3:** flexural properties of rWTB composites.

Sample formulation			Max load (lb)	MOR (MPa)	Slope	MOE (MPa)
MDI(%)	MC(%)	MSS(mm)				
10	5	12.7	125.5	36.99423	603.35	5290.156
10	5	12.7	174	52.10581	663.9732	5967.586
10	5	12.7	126.5	35.79388	581.3805	4803.297
10	5	12.7	126.6	39.32278	527.2857	5001.69
10	5	12.7	143.5	43.95182	560.0405	5210.917
10	5	12.7	127.2	36.49764	520.3024	4388.266
3	5	12.7	51.17	16.54371	293.3341	2956.343
3	5	12.7	72.21	20.44546	432.7902	3577.026
3	5	12.7	82.58	23.45193	493.5683	4097.078
3	5	12.7	88.16	24.0843	483.7854	3788.442
3	5	12.7	89.66	23.19573	461.6024	3329.199
3	5	12.7	72.48	19.21738	420.9317	3152.203
6	3	12.7	100.7	30.55133	459.585	4212.482
6	3	12.7	107	31.99349	484.4756	4346.526
6	3	12.7	84.03	23.18516	470.0683	3730.436
6	3	12.7	107	29.031	603.6268	4674.992
6	3	12.7	119.2	32.1301	573.4134	4393.735
6	3	12.7	96.59	26.45671	502.3829	3944.429
6	8	12.7	127.7	37.03997	526.9293	4507.024
6	8	12.7	122.9	35.85563	557.1439	4807.468
6	8	12.7	117.2	35.80041	523.8171	4835.387
6	8	12.7	78.34	25.42482	342.378	3463.818
6	8	12.7	104.6	35.01501	478.7805	5078.366
6	5	3.18	120	33.40345	505.5707	4069.093
6	5	3.18	102	30.00319	496.1829	4337.754
6	5	3.18	88.59	25.80307	529.7878	4552.814
6	5	3.18	107.8	31.20296	550.7024	4686.729
6	5	3.18	99.01	28.62836	508.8878	4321.655
6	5	3.18	75.87	22.14576	482.8146	4159.192
6	5	0.25	98.09	31.75719	421.4712	4258.472
6	5	0.25	90.63	27.60545	456.7051	4213.159
6	5	0.25	91.81	25.59409	472.2073	3806.169
6	5	0.25	103.5	28.7969	474.0683	3814.753
6	5	0.25	105.4	28.55515	526.6683	4070.888
6	5	0.25	103.8	28.67821	468.122	3728.742
6	5	0.125	100.8	27.50151	518.9833	4055.596
6	5	0.125	83.65	22.41048	467.9405	3558.208
6	5	0.125	83.7	25.27785	431.4214	3909.388
6	5	0.125	89.6	27.47163	434.1857	4035.16
6	5	0.125	79.25	25.01151	393.0964	3814.264
6	5	0.125	86.6	27.85982	420.4976	4200.247

**Table 4:** IB test results of rWTB composites.

Sample formulation			Max Load (lbs)	IB (Psi)
MDI (%)	MC(%)	MSS(mm)		
10	5	12.7	1071	267.592
10	5	12.7	1384	345.6629
10	5	12.7	1429	358.8049
10	5	12.7	1457	366.1318
10	5	12.7	1439	371.2913
3	5	12.7	564.8	140.0127
3	5	12.7	553	138.0326
3	5	12.7	610.7	152.8257
3	5	12.7	292.2	73.03622
3	5	12.7	374.3	93.63283
6	3	12.7	1100	273.5979
6	3	12.7	662.8	165.6675
6	3	12.7	1003	251.2946
6	3	12.7	1014	253.5499
6	3	12.7	751.1	188.7062
6	8	12.7	1212	303.4863
6	8	12.7	1038	261.3079
6	8	12.7	929.4	234.2984
6	8	12.7	983.1	248.2317
6	8	12.7	1104	278.8125
6	5	3.18	763.8	189.7901
6	5	3.18	1191	296.1163
6	5	3.18	886.4	220.6874
6	5	3.18	1279	318.1838
6	5	3.18	900.4	224.5251
6	5	0.25	1097	271.9967
6	5	0.25	1170	291.7529
6	5	0.25	1199	299.2785
6	5	0.25	1620	403.7277
6	5	0.25	1253	311.4083
6	5	0.125	1019	255.1517
6	5	0.125	766.2	192.0794
6	5	0.125	1254	314.1181
6	5	0.125	1052	263.3628
6	5	0.125	1152	288.4544
