# Supplementary material for: Data on the mechanical properties of recycled wind turbine blade composites
Source: Data Brief. 2018 May 9;19:230–5. doi: 10.1016/j.dib.2018.05.008 (PMC5992999; doi:10.1016/j.dib.2018.05.008)
Supplement: Supplementary file 1 — Supplementary material [file mmc1.docx]

**Declaration of Conflicting Interests**

The author(s) declared no potential conflicts of interest with respect to the research, authorship, and/or publication of this article.
